# Help-seeking behaviour among people living with chronic hip or knee pain in the community

**DOI:** 10.1186/1471-2474-10-153

**Published:** 2009-12-07

**Authors:** Carina A Thorstensson, Rachael Gooberman-Hill, Joy Adamson, Susan Williams, Paul Dieppe

**Affiliations:** 1 Research and Development Centre, Spenshult Hospital for Rheumatic Diseases, Oskarström, Sweden; 2 Clinical Science at North Bristol/Department of Social Medicine, University of Bristol, UK; 3 Department of Health Sciences, University of York, UK; 4 Peninsula School of Medicine, Universities of Exeter and Plymouth, UK

## Abstract

**Background:**

A large proportion of people living with hip or knee pain do not consult health care professionals. Pain severity is often believed to be the main reason for help seeking in this population; however the evidence for this is contradictory. This study explores the importance of several potential risk factors on help seeking across different practitioner groups, among adults living with chronic hip or knee pain in a large community sample.

**Methods:**

Health care utilization, defined as having seen a family doctor (GP) during the past 12 months; or an allied health professional (AHP) or alternative therapist during the past 3 months, was assessed in a community based sample aged 35 or over and reporting pain in hip or knee. Adjusted odds ratios were determined for social deprivation, rurality, pain severity, mobility, anxiety/depression, co-morbidities, and body mass index.

**Results:**

Of 1119 persons reporting hip or knee pain, 52% had pain in both sites.

Twenty-five percent of them had seen a doctor only, 3% an AHP only, and 4% an alternative therapist only. Thirteen percent had seen more than one category of health care professionals, and 55% had not seen any health care professional. In the multivariate model, factors associated with consulting a GP were mobility problems (OR 2.62 (1.64-4.17)), urban living (OR 2.40 (1.14-5.04) and pain severity (1.28 (1.13-1.44)). There was also some evidence that obesity was associated with increased consultation (OR 1.72 (1.00-2.93)). Factors were similar for consultation with a combination of several health care professionals. In contrast, seeing an alternative therapist was negatively associated with pain severity, anxiety and mobility problems (adjusting for age and sex).

**Conclusion:**

Disability appears to be a more important determinant of help-seeking than pain severity or anxiety and depression, for adults with chronic pain in hip or knee. The determinants of seeking help from alternative practitioners are different from determinants of consulting GPs, AHPs or a combination of different health care providers.

## Background

Chronic hip and knee pain are common amongst older people in the community and are a major cause of disability [1-5]. The main cause is osteoarthritis, and as there is no definitive treatment for this group of disorders, management is purely symptomatic. However, previous research indicates that a large proportion of individuals with these symptoms do not consult health care professionals [6], despite the availability of a range of treatment options [7-9].

In order to understand this, some attempts have been made to identify the factors associated with consultation for hip and knee pain. Pain severity is often believed to be the main reason for help seeking in this population, however the evidence for this is contradictory. For example, recent studies have revealed that severe pain and greater self-reported disability were not associated with consultation of health care professionals [6,10]. Several other factors have been associated with help-seeking for joint pain, including socio-demographic characteristics, psychological factors and co-morbidity [6,10-12]; for example, previous work has suggested that anxiety and depression have a negative effect on help-seeking behaviour [10]. However, the findings are not conclusive. Some of the variation between studies will be explained by methodological issues including the definitions of help-seeking and of joint pain. Most studies define help-seeking as consultation with any health practitioner, rather than separating out the different kinds of practitioners used and definitions of joint pain do not always differentiate between acute and chronic conditions. This study, therefore, examines the importance of several potential risk factors on help seeking across *different practitioner groups*, among people living with *chronic *hip or knee pain in a large community sample.

## Methods

Ethical approval for the baseline study was received from five local NHS research ethics committees; for the follow-up study approval was obtained from the South West Multi-centre Research Ethics Committee. Written informed consent was obtained according to the Declaration of Helsinki (Br Med J 1996;31:1448-9) from all participants in the study reported here.

**Figure 1:**
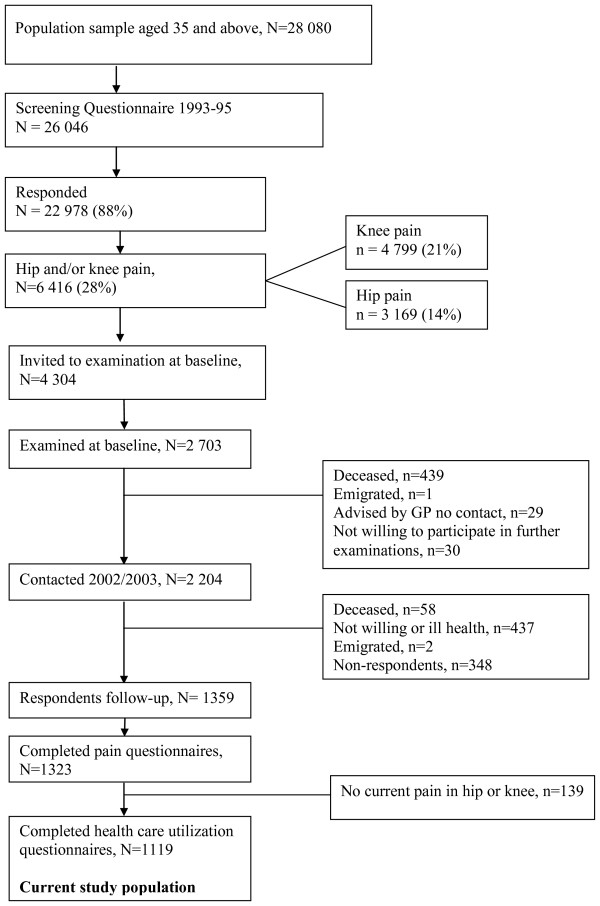
**Flow chart of study participants**.

The data are derived from the follow-up of a cohort derived from a community-based study, the 'Somerset and Avon Survey of Health' (SASH), which has been described previously [13,14]. In brief, contact details of a representative sample of 28,080 urban or rural dwelling adults, aged 35 and over, were obtained from the lists of 40 general practices in the South West of England. A screening questionnaire for pain in hips or knees was sent out to 26,046 of these people in 1994/1995. The response rate was 88%. Overall 6,416 persons (28%) reported pain in or around either the hip and/or knee on most days for one month or longer during the past year. A geographically and age representative sample of 4304 people with hip or knee pain was invited to examination, and 2,703 (63%) attended. A second questionnaire was sent out to this group, on average 7.5 years later, with an invitation to attend for a second interview and clinical examination. One thousand three hundred and fifty-nine persons (50%) responded to the second questionnaire in 2002/3, and questions on current pain in hips or knees were completed by 1,323 persons (Figure 1).

### Outcome measure

The main outcome used for this study was self-reported health care utilization at follow-up. The use of health care was defined as having seen a family doctor (GP) for hip or knee pain during the past 12 months, or as having seen an allied health professional (AHP) or alternative therapist during the past 3 months for the hip or knee pain. The allied health professionals specified in the questionnaire were nurse, physiotherapist or occupational therapist. Complementary or alternative therapists could be homeopath, acupuncturist, osteopath, aromatherapist, chiropractor or any other alternative therapist as defined by participants. If a respondent had not seen any of these health care professionals during the past 12 or 3 months respectively, the respondent was classified as "not a current user". To avoid bias from including individuals more than once in analyses, persons who had consulted more than one of the three categories of health care professionals were categorised as having seen a combination of care givers.

### Explanatory Variables

Data were examined in order to explain any observed differences in utilization of the various types of health care professional by people in the survey. Age, gender, and ethnicity were recorded, and body mass index (BMI) data was obtained at baseline. BMI was grouped into four categories, using the World Health Organization standards; underweight equals BMI < 18.50 kg/m^2^; normal weight 18.50 - 24.99 kg/m^2^; overweight 25.00 - 29.99 kg/m^2^; obesity BMI ≥ 30.00 kg/m^2 ^[15]. To estimate social deprivation, the postcodes of all respondents were linked to enumeration districts, and the Townsend deprivation score was derived [16]. The rurality variable has been created from census area statistics (CAS) at ward level. Postcodes were used to identify the ward of residence, using the Office for National Statistics all fields postcode directory. Participants rated pain severity for right and left hip and knee at follow up on four separate 1-10 Likert-style scales, where 1 represented no pain in or around hip or knee over the past 12 months, and 10 corresponded to pain as bad as it could be, even if taking medications. An average score for hips and knees was calculated by summing the separate scores for both hips and knees and dividing by 4. The modified score of 1 thus represents "no pain" in either hip or knee, and 10 represents "pain as bad as it can be" in both hips and both knees. To estimate the impact of physical function and mental health on use of health care, the mobility and anxiety/depression subscales from the Euroqol 5 dimensions (EQ5D) were used [17]. Participants stated their current physical function on a 3-graded scale; no problems walking about, some problems walking about, or confined to bed, and anxiety/depression was graded as not anxious or depressed, moderately anxious or depressed, or extremely anxious or depressed.

Comorbidities were classified as the number of self-reported health problems. Participants were asked to state if a doctor had ever told them they had any of a group of predefined conditions. The health problem areas were categorised into six groups; 1) arthritis (osteoarthritis, rheumatoid arthritis, rheumatism or other arthritis); 2) heart trouble (angina, heart failure, heart attack or other heart trouble); 3) chest trouble (asthma, emphysema, bronchitis, other chest trouble); 4) eye trouble (cataract, glaucoma, diabetic eye disease, other eye trouble); 5) circulatory trouble (high blood pressure, deep vein thrombosis, intermittent claudication); and 6) other health problems (stroke, diabetes, depression, cancer or any other condition). The validity of these screening questions in this population has been described previously [14], showing specificity over 90% for all conditions except arthritis (79%). The sensitivity was more varied, and ranged from 50 to 70%, except for bronchitis and depression where the sensitivity was about 30%. For the present study health problem areas were classified in four categories according to the number of reported problems: 0-1, 2, 3, and 4 or more other health problem areas.

### Data handling and statistics

Univariate logistic regression was used to determine the importance of age and gender for the use of health care. All other variables were adjusted for age and gender. To minimize the influence of missing data the 190 cases with missing BMI data were grouped into a separate category and included in the model. Omitting the "missing" values from the model did not affect the outcome. Due to low numbers in the "underweight" category of BMI (n = 3), the mobility category "confined to bed" (n = 6), and the extremely anxious or depressed category (n = 26), these categories were collapsed with the closest alternative. Underweight was collapsed with normal weight, confined to bed was collapsed with "some mobility problems" to a new category called mobility problems, and the category extremely anxious or depressed was combined with "moderately anxious or depressed" to a new category called anxious or depressed. Multivariate logistic regression, adjusting for all variables in the univariate analysis, was conducted for the subgroups of people seeking care from a GP and a combination of health care providers. Due to low numbers, such analysis could not be performed for the AHP and alternative practitioners groups. All statistics were derived using SPSS 16.0.

## Results

### Study population

The overall response rate at follow-up was 62% of those known to be alive (Figure 1). At follow-up, the pain questionnaire was completed by 1323 participants, of whom 363 (27%) had pain in knees only, 137 (10%) had pain in hips only, and 684 (52%) had pain in both hips and knees. One hundred and thirty nine persons (11%) no longer had pain in either the hip or the knee. They were excluded from further analyses, since the aim of the present study was to explore health care utilization among people who experience long-standing pain in hips or knees. Health care utilization data was completed by 1119 of the participants with hip or knee pain, representing 41% of those examined 7.5 years earlier, and 49% of those known to be alive at the time of the second examination (Figure 1). This constituted the current study population. These 1119 people had a mean age of 67.7 years (SD 11.0, range 42-98), and 62% were women (see Additional file 1: Table including baseline descriptives). Note that the study population differs from that reported in Ayis et al [13] because the participants included here are those who completed health care utilization and pain items, whereas Ayis and colleagues employed data from participants about whom there was self-reported data on walking ability plus examination data.

### Health care utilization

**Figure 2:**
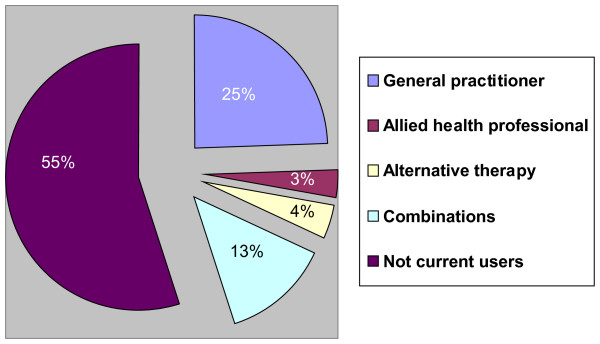
**Percentage of people with hip and/or knee pain who have seen a health care professional for their hip and knee problems (Combinations includes all different combinations of health care providers)**. N = 1119.

Two hundred and seventy-five persons (25%) had seen only a GP during the past 12 months for their hip or knee pain. During the past 3 months, 36 persons (3%) had visited an allied health professional (AHP) without seeing the GP during the past year. Another 118 persons (10%) had seen an AHP in combination with a GP and/or an alternative therapist. An alternative therapist had been seen by 45 persons (4%) without a visit to a GP during the past year, and another 58 (5%) had seen an alternative therapist in combination with a GP and/or an AHP. Of the 147 (13%) who had seen more than one category of health care professionals all but 13 had seen a GP. Six hundred and sixteen persons (55%) were categorized as "not a current user" of health care professionals (Figure 2). For the purposes of analysing the explanatory variables we have split the groups in the four different categories of health care use as shown in Additional file 1. Fifty percent or more of the persons across all groups were overweight or obese. About 80% of the persons who had visited a GP, AHP or a combination of several health care providers had some mobility problems, and about two thirds had pain in both hip and knee. In the group who had visited an alternative therapist 40% experienced mobility problems and 49% had pain in both hip and knee (see Additional file 1: Table including baseline descriptives).

### Explanatory variables

Table 1 shows the odds ratios for the association of various demographic factors with the four different health care utilization groups described above.

**Table 1:** Influence of demographic and socioeconomic factors on seeking care for hip or knee pain.

	GP*	AHP*	Alt ther*	Combinations*
	**OR (95% CI)^†^**	**OR (95% CI)^†^**	**OR (95% CI)^†^**	**OR (95% CI)^†^**

**Age**	1.02 (1.00-1.03)	1.01 (0.98-1.05)	0.98 (0.96-1.01)	1.01 (1.00-1.03)

**Man**	1.00	1.00	1.00	1.00

**Woman**	1.28 (0.95-1.72)	1.10 (0.55-2.18)	2.44 (1.19-5.02)	1.20 (0.83-1.74)

**Normal weight (BMI < 25 kg/m^2^)**	1.00	1.00	1.00	1.00

**Overweight (BMI 25-29.99 kg/m^2^)**	1.66 (1.11-2.48)	1.27 (0.52-3.10)	2.24 (1.01-4.95)	1.51 (0.93-2.43)

**Obese (BMI ≥ 30 kg/m^2^)**	2.47 (1.63-3.74)	1.01 (0.35-2.91)	1.38 (0.54-3.49)	1.69 (1.01-2.84)

**Lowest quintile of deprivation**	1.00	1.00	1.00	1.00

**Highest quintile of deprivation**	1.43 (0.93-2.20)	0.84 (0.28-2.54)	0.40 (0.13-1.84)	1.26 (0.75-2.12)

**Living in rural areas**	1.00	1.00	1.00	1.00

**Living in urban areas**	1.78 (1.11-2.88)	1.37 (0.47-3.96)	0.72 (0.32-1.62)	1.08 (0.64-1.82)

**Pain in both hips and knees**	1.00	1.00	1.00	1.00

**Pain in hips only**	0.52 (0.31-0.87)	0.54 (0.16-1.86)	1.73 (0.76-3.93)	0.60 (0.32-1.13)

**Pain in knees only**	0.57 (0.41-0.80)	0.57 (0.26-1.26)	1.11 (0.55-2.24)	0.65 (0.43-0.99)

**Pain severity**	1.44 (1.32-1.58)	1.32 (1.09-1.61)	0.78 (0.61-1.01)	1.51 (1.36-1.68)

**Not anxious/depressed**	1.00	1.00	1.00	1.00

**Anxious/depressed**	1.61 (1.20-2.17)	1.84 (0.93-3.65)	0.84 (0.43-1.65)	1.53 (1.05-2.20)

**No mobility problems**	1.00	1.00	1.00	1.00

**Mobility problems**	3.83 (2.72-5.39)	4.72 (1.92-11.62)	0.69 (0.37-1.29)	3.87 (2.49-6.01)

**0-1 other health problems**	1.00	1.00	1.00	1.00

**2 health problem areas**	1.74 (1.08-2.81)	1.33 (0.41-4.35)	2.53 (1.00-6.36)	2.17 (1.14-4.11)

**3 health problem areas**	1.93 (1.21-3.08)	1.06 (0.31-3.58)	1.38 (0.50-3.77)	1.90 (1.00-3.62)

**4 or more health problem areas**	1.76 (1.10-2.83)	2.27 (0.77-6.68)	1.47 (0.54-4.03)	2.60 (1.40-4.85)

*Health care professionals are divided into general practitioners (GP), allied health professionals (AHP), alternative therapists (alt ther) or a combination of two or more categories.

^†^Univariate odds ratios (OR) and 95% confidence intervals (CI) are given for age and gender. All other variables are age and gender adjusted.

Looking first at the group who had visited a GP in the last year to consult about hip or knee pain, it is clear that the strongest determinants were mobility problems (OR 3.83 (2.72-5.39)), obesity (2.47 (1.63-3.74)) and 3 or more other health problems (1.93 (1.21-3.08)). Significant associations, but with smaller odds ratios, were also seen with urban living (1.78 (1.11-2.88)), anxiety/depression (1.61(1.20-2.17)) and pain severity (1.44 (1.32-1.58)).

The pattern of the associations was very similar in the 'combinations' group, with mobility problems (3.87 (2.49-6.01)) and co-morbidities (2.60 (1.40-4.85)) having the highest odds ratios, followed by obesity (1.69 (1.01-2.84)), anxiety/depression (1.53 (1.05-2.20)) and pain severity (1.51 (1.36-1.68)) in that order.

The other two groups (consulting an allied health professionals or an alternative therapist within the last three months) are smaller, and fewer of the associations reached statistical significance therefore. However, it is clear that the pattern of associations for those who visited allied health professionals is similar to that for GPs or the 'combinations' group, the highest odds ratios being for mobility problems (4.72), co-morbidities (2.27), anxiety/depression (1.84), living in urban areas (1.37), pain severity (1.32) and overweight (1.27) in that order. In contrast, the pattern for those who had recently visited an alternative practitioner is completely different. This is the only group in which a gender association was apparent, there being a female preponderance. There was some association with being overweight (2.24) or having co-morbidities (2.53), as in other groups, but many of the other demographic factors that were positively associated with the other three groups showed a negative association with visits to alternative therapists. Deprivation (0.40) and living in an urban area (0.72) both showed a negative rather than positive association in this group, as did anxiety/depression (0.84), pain severity (0.78) and mobility problems (0.69) - the factors with the highest positive odds ratio association with the other three groups.

In the multivariate analysis, taking all variables into account, mobility problems still showed the strongest association with seeing a GP (OR 2.62 (95% CI 1.64-4.17)). Pain severity also showed an association, albeit weaker (1.28 (1.13-1.44)), while living in urban areas showed stronger association compared to the age and gender adjusted model (2.40 (1.14-5.04)). Having two or three, but not four, co-morbidities still had higher odds ratios than pain severity; however the statistical significance was lost adjusting for all other variables (Table 2). Seeing a combination of health care providers was associated with mobility problems (2.77 (1.83-4.20)), being overweight (1.71 (1.09-2.68)) or obese (1.62 (1.00-2.62)), and with pain severity (1.48 (1.34-1.64)). Anxiety/depression showed no association with seeking care from GP or a combination of health care professionals in the multivariate analysis (Table 2).

**Table 2:** Multivariate model of influence of demographic and socioeconomic factors on seeking care for hip or knee pain.

	GP*	Combinations*
	**OR (95% CI)^†^**	**OR (95% CI)^†^**

**Age**	1.00 (0.99-1.02)	1.00 (0.98-1.02)

**Man**	1.00	1.00

**Woman**	1.24 (0.84-1.85)	1.17 (0.83-1.65)

**Normal weight (BMI < 25 kg/m^2^)**	1.00	1.00

**Overweight (BMI 25-29.99 kg/m^2^)**	1.35 (0.81-2.26)	1.71 (1.09-2.68)

**Obese (BMI ≥ 30 kg/m^2^)**	1.72 (1.00-2.93)	1.62 (1.00-2.62)

**Lowest quintile of deprivation**	1.00	1.00

**Highest quintile of deprivation**	1.00 (0.57-1.74)	0.98 (0.59-1.61)

**Living in rural areas**	1.00	1.00

**Living in urban areas**	2.40 (1.14-5.04)	1.18 (0.71-1.97)

**Pain in both hips and knees**	1.00	1.00

**Pain in hips only**	0.96 (0.56-1.66)	0.96 (0.59-1.57)

**Pain in knees only**	1.03 (0.67-1.59)	1.17 (0.80-1.72)

**Pain severity**	1.28 (1.13-1.44)	1.48 (1.34-1.64)

**Not anxious/depressed**	1.00	1.00

**Anxious/depressed**	0.88 (0.58-1.33)	1.04 (0.73-1.49)

**No mobility problems**	1.00	1.00

**Mobility problems**	2.62 (1.64-4.17)	2.77 (1.83-4.20)

**0-1 other health problems**	1.00	1.00

**2 health problem areas**	1.60 (0.84-3.05)	1.31 (0.76-2.28)

**3 health problem areas**	1.76 (0.93-3.34)	1.15 (0.66-2.01)

**4 or more health problem areas**	1.28 (0.66-2.48)	1.26 (0.73-2.18)

*Health care professionals are divided into general practitioners (GP), or any combination of general practitioners, allied health professionals, and alternative therapists.

^†^All variables are included in the model. Adjusted odds ratios (OR) and 95% confidence intervals (CI)

## Discussion

Among a population of community-based adults with chronic hip and knee pain, the majority had not sought help from their GPs over the last 12 months. The strongest determinants of seeking help from GPs or allied health professionals for this pain were reduced mobility, living in urban areas, pain severity and obesity. The presence of co-morbidities, anxiety/depression and socio-demographic characteristics were of much less importance. A further important finding was that only a minority of the group reported pain that was confined to the hips or the knees, the majority having pain at both joint sites. In addition the data suggest that people seek help from alternative practitioners for quite different reasons, with pain severity, anxiety and mobility problems showing negative associations. The demographic data also indicate that this subgroup differs from the other help-seeking groups (see Additional file 1: Table including baseline descriptives).

Those respondents who reported difficulty walking were more likely to consult their GP, allied health care professionals and a combination of health care providers, than those whose walking was unimpaired. Even controlling for all other variables, mobility problems was still the strongest determinant of help-seeking. This is in agreement with findings from others where disabling chronic pain was more associated with care use than chronic pain with little or no pain-related disability [18]. This indicates that it may be more important to consider assessments of walking ability than assessments of pain in this population with chronic hip or knee pain.

Some previous data has suggested that pain severity is one of the key determinants of help-seeking behaviour and that anxiety or depression can have a negative impact [10,12]. Our results indicate that pain severity is important, but not nearly as influential as mobility difficulties. In the age and gender adjusted analysis co-morbidities or being overweight or obese showed stronger association with help seeking than pain severity. This was true for seeing a GP in the multivariate analysis also, albeit the odds ratios did not reach statistical significance. Being overweight or obese also showed stronger association with seeking a combination of health care professionals than pain severity even controlled for all other variables. The findings from the analysis do not support the previous suggestion that anxiety/depression have a negative impact on consultation [10]. The age and gender adjusted analysis indicating that people reporting higher levels of anxiety or depression consulted a GP or a combination of health care providers *more *often than those who did not, however, these associations were attenuated in the multivariate model indicating anxiety and depression had little impact on consultation.

Localised joint pain alone seems to be of less importance than joint pain at both the hip and knee, or joint pain in combination with other health problems. The fact that more people in this community-based study had a combination of hip and knee pain than pain in one site alone suggests that it may be more appropriate to consider 'lower-limb pain' as the common problem rather than osteoarthritis confined to hip or knee joints.

Multiple health problems are very common at older ages [19]. Whilst it has previously been shown that seeking help for comorbidities does not increase levels of consultation for knee pain [20], in initial analysis, we found a positive association between number of comorbidities and consultation with a GP and combination of health care professionals, however these associations were attenuated in the multivariate model.

Living in urban areas increased the probability of consulting a GP, and the association remained strong adjusting for all other variables. This has also been demonstrated in a study comparing help-seeking for mental health in urban and rural populations, without showing any differences in perceived health status [21].

Fifty-five percent of the people living with long-term hip or knee pain in the community did not consult a GP in the previous 12 months or an AHP in the past 3 months. There are several possible reasons for this, for example, previous studies have shown that perceived need is influenced by beliefs that joint pain is an inevitable process and that there is nothing much that can be done about it [6,11,22,23]. It is also possible that individuals who are not currently seeking care may have consulted these services previously, and not found any need for a further consultation. We are not able to explore the importance of people's beliefs about the usefulness of health care for hip or knee pain, however, this may be an important factor for current help-seeking. Furthermore, it is possible that use of health care at baseline could determine future help-seeking behaviour and use of health care. Baseline health care utilisation was recorded for the population as a whole. However, for the 1 119 persons who constituted this study cohort, the baseline health care utilisation data were incomplete in a large number of cases, which prevented us from addressing this question. The use of other strategies to manage joint pain, like self-management and over the counter drugs, has not been included in this study.

The study has highlighted several factors that are associated with consultation across different groups of health care providers for people with long-term hip and knee pain in the community. Whilst we have focused on socio-demographic and symptom based triggers for consultation, it is important to highlight that reviews of the literature on consultation with formal health services have demonstrated the complexity of the decision to seek help. Socio-demographic characteristics and symptoms are only part of this picture which is also influenced by a range of other psycho-social and access factors [24], which we are not able to consider here.

The pattern of associations amongst those who sought help from alternative practitioners was quite different from that of consultations with GPs, AHPs or a combination of health care providers. It has previously been suggested that people who primarily rely on alternative medicine are less satisfied with conventional medicine compared to people who use complementary medicine in conjunction with conventional care [25]. It seems likely that people are using alternative practitioners for different reasons and with different expectations from those that they take to their GP or AHP. Although not many of the associations reached statistical significance, because of relatively small numbers, the data suggest a preponderance of women in this group, which is in line with findings from others [26,27]. We also found that many of the factors affecting the decision to go to a GP or AHP, such as mobility restriction, pain severity, and anxiety/depression, are not determining the decision to go to an alternative practitioner.

### Strengths and limitations

Strengths of this study include the large sample size and its being community based. The original response rate was high (86%) in this large, population-based cohort. The overall response rate at follow-up was 62% of the population known to be alive. This, together with a sample representative for the adult population living in the community, increases the possibility of generalising the results to a larger population. In addition, the amount of missing data was small. Weaknesses include the reliance on self-report data with regard to help-seeking behaviours and co-morbidities which may mean that there is some recall bias. In addition, data comprised different time spans regarding help seeking from a GP (last 12 months) compared to AHPs or alternative therapists (last three months). This design decision means that reports of AHP or alternative therapists use are likely to be more reliable than those about GPs, but a longer time span for the AHP and alternative therapist data might have provided more data. The smaller numbers in these groups may have limited our ability to find statistically significant associations, and also prevented us from performing multivariate logistic regression analyses in these groups. Finally, using BMI at baseline only means that is not possible to ascertain how weight loss or gain during the years to follow-up might affect help-seeking behaviour.

## Conclusion

This study supports previous data that indicate that many adults with chronic musculoskeletal pain do not seek help from health care professionals. New findings in this study are that disability is a more important determinant of help-seeking than pain severity, that co-morbidities and overweight/obesity are also important determinants, and that anxiety and depression are not negatively associated as has been suggested previously. In addition our findings suggest that the use of alternative practitioners is related to reasons other than pain severity and mobility problems, however the determinants for seeing alternative therapists need to be further explored.

## Competing interests

The authors declare that they have no competing interests.

## Authors' contributions

CT participated in study design, data analysis and writing of the manuscript. RG-H, JA, SW, and PD participated in study design, interpretation of data, and critically revised the manuscript. All authors read and approved the final manuscript.

## Pre-publication history

The pre-publication history for this paper can be accessed here:

http://www.biomedcentral.com/1471-2474/10/153/prepub

## Supplementary Material

Additional file 1 **Table including baseline descriptives**. Descriptives for not current users and different groups of users of health care for hip and knee pain. Click here for file
