# Supplementary material for: Help-seeking behaviour among people living with chronic hip or knee pain in the community
Source: BMC Musculoskelet Disord. 2009 Dec 7;10:153. doi: 10.1186/1471-2474-10-153 (PMC2793247; doi:10.1186/1471-2474-10-153)
Supplement: Additional file 1 — Table including baseline descriptives. Descriptives for not current users and different groups of users of health care for hip and knee pain. [file 1471-2474-10-153-S1.DOC]

Descriptives for not current users and different groups of users of health care for hip and knee pain.

|  | ***Not current user  n=616*** | ***GP,***  ***N=275*** | ***AHP***  ***N=36*** | ***Alt ther***  ***N=45*** | ***Combinations***  ***N=147*** | ***Total n=1119*** |
| --- | --- | --- | --- | --- | --- | --- |
| **Age, mean (SD)** | 67.0 (11.1) | 68.9 (10.5) | 68.7 (11.8) | 64.7 (9.9) | 68.4 (11.6) | 67.6 (11.0) |
| **Women, n (%)** | 363 (59) | 178 (65) | 22 (61) | 35 (78) | 93 (63) | 691 (62) |
| **White-Caucasian, n (%)** | 607 (98) | 269 (98) | 34 (94) | 43 (96) | 143 (97) | 1096 (99) |
| **Underweight (< 18.5 kg/m^2^), n (%) *** | 3 (0.5) | - | - | - | - | 3 (0.3) |
| **Normal weight (18.50-24.99 kg/m^2^), n (%) *** | 189 (31) | 51 (18) | 9 (25) | 10 (22) | 34 (23) | 293 (26) |
| **Overweight (25.00-29.99 kg/m^2^), n (%) *** | 198 (32) | 88 (32) | 12 (33) | 20 (44) | 53 (36) | 371 (33) |
| **Obese (≥30 kg/m^2^), n (%) *** | 126 (20) | 83 (30) | 6 (17) | 9 (20) | 38 (26) | 262 (23) |
| **Least deprived quintile, n (%)** | 160 (26) | 70 (26) | 10 (28) | 10 (22) | 44 (30) | 294 (26) |
| **Most deprived quintile, n (%)** | 97 (16) | 59 (22) | 5 (14) | 3 (7) | 33 (22) | 197 (18) |
| **Rural, n (%)** | 88 (14) | 24 (9) | 4 (11) | 8 (18) | 20 (14) | 144 (13) |
| **Pain in hips only, n (%)** | 77 (12) | 22 (8) | 3 (8) | 9 (20) | 13 (9) | 124 (11) |
| **Pain in knees only, n (%)** | 210 (34) | 69 (25) | 9 (25) | 14 (31) | 40 (27) | 342 (31) |
| **Pain in hips and knees, n (%)** | 329 (53) | 184 (67) | 24 (67) | 22 (49) | 94 (64) | 653 (58) |
| **Total pain intensity hip and knee,  mean (SD) (1-10, best – worst)** | 3.1 (1.6) | 4.0 (1.6) | 3.5 (1.6) | 2.8 (1.0) | 4.5 (2.0) | 3.6 (1.8) |
| **No mobility problems, n (%)** | 301 (49) | 54 (20) | 6 (17) | 27 (60) | 29 (20) | 417 (37) |
| **No anxiety/depression, n (%)** | 428 (70) | 161 (58) | 20 (56) | 32 (71) | 88 (60) | 729 (65) |
| **0-1 other health problems, n (%)** | 138 (23) | 35 (13) | 5 (15) | 7 (16) | 16 (11) | 201 (19) |
| **2 other health problem areas, n (%)** | 138 (23) | 65 (25) | 7 (21) | 17 (38) | 36 (26) | 263 (25) |
| **3 other health problem areas, n (%)** | 148 (25) | 78 (30) | 6 (18) | 10 (22) | 34 (24) | 276 (26) |
| **4 or more other health problem areas, n (%)** | 167 (28) | 84 (32) | 15 (46) | 11 (24) | 54 (39) | 331 (31) |

^*^ Missing values on BMI for 190 cases (100 no current users, 53 GP, 9 AHP, 6 alternative therapist, 22 combination of health care professionals)
